# Risk stratification for stroke in acute persistent vertigo: development and internal validation of a multivariable prediction model

**DOI:** 10.3389/fneur.2026.1822762

**Published:** 2026-06-24

**Authors:** Yue Ba, Bin Liu, Pinyi Zhu

**Affiliations:** Department of Geriatrics, Affiliated Brain Hospital of Nanjing Medical University, Nanjing, China

**Keywords:** acute persistent vertigo, clinical decision-making, internal validation, LASSO logistic regression, multivariable prediction model, neurological risk assessment, risk tertiles, stroke risk stratification

## Abstract

**Background:**

Despite the fact that acute persistent vertigo can be a presenting sign of stroke, early bedside risk assessment continues to be particularly difficult. In order to predict clinically detected strokes among patients who were experiencing acute persistent vertigo, our objective was to create and internally validate a multivariable model. Additionally, we wanted to evaluate the model’s performance in comparison to scores that are routinely utilized.

**Methods:**

We retrospectively analyzed 689 consecutive patients with acute persistent vertigo, operationally defined as vertigo lasting longer than 24 h, including 165 clinically diagnosed strokes (23.9%). Candidate predictors were prespecified and selected using LASSO-regularized logistic regression, followed by multivariable logistic modeling. Internal validation was performed using 5-fold cross-validation, with 95% confidence intervals obtained via bootstrap resampling (*n* = 1,200). Discrimination (AUC), calibration (intercept and slope), Brier score, decision-curve analysis, and risk stratification by tertiles of predicted probability were assessed and compared with ABCD2, CNS, and Triage-Plus scores.

**Results:**

The whole cohort had a mean age of 60.3 ± 12.9 years and included 376 males (54.6%) and 313 females (45.4%); the stroke subcohort had a mean age of 65.0 ± 14.6 years and included 89 males (53.9%) and 76 females (46.1%). In the multivariable model, older age (analyzed as a continuous variable; OR 1.050 per 1-year increase), smoking (OR 2.147), hypertension (OR 5.452), hyperlipidemia (OR 2.621), diabetes mellitus (OR 3.918), coronary heart disease (OR 4.361), history of atrial fibrillation (OR 10.376), higher CNS score (OR 1.679 per point), and nausea/vomiting (OR 2.020) were associated with increased stroke odds, whereas tinnitus was inversely associated (OR 0.382). The model showed excellent discrimination (AUC 0.902; 95% CI 0.874–0.927), with sensitivity 0.873 and specificity 0.792 at the Youden threshold, good calibration (intercept −0.036; slope 0.973), and a low Brier score (0.096). Performance exceeded ABCD2 (AUC 0.642) and Triage-Plus (AUC 0.514), and was higher than CNS alone (AUC 0.846). Risk tertiles (cut-points 0.039 and 0.242) yielded stroke event rates of 2.6, 10.5, and 58.7% in low-, intermediate-, and high-risk groups, respectively.

**Conclusion:**

Our findings suggest that a multivariable prediction model may assist in risk stratification and diagnostic decision-making, as well as providing tailored stroke risk assessment for acute persistent vertigo. It would be beneficial to perform impact studies and external validation.

## Introduction

Within the realm of clinical practice, acute persistent vertigo is a symptom that is frequently encountered. This symptom frequently presents diagnostic issues due to the vast variety of probable causes that it can have. Cerebrovascular events, which include strokes, are among these, and they are a substantial cause that is generally underestimated (1–3). It is extremely important to detect strokes in patients who appear with acute vertigo as soon as possible because this can have a considerable impact on the outcomes of the patient, particularly with regard to the treatment options and the prognosis to be considered. In spite of the fact that this link is clinically significant, doing effective and timely stroke risk stratification continues to be difficult. This is partly because there are no clear guidelines that are generally acknowledged (4, 5).

For the purpose of assisting in the diagnosis of stroke in patients who are experiencing acute vertigo or dizziness, a number of clinical scoring systems have been proposed. The ABCD2 score, the CNS score, and the Triage-Plus score are all examples of their respective scores (1, 3). Although these instruments have demonstrated their usefulness in various clinical scenarios, when applied to the acute vertigo cohort, they frequently fail to meet the required levels of sensitivity and specificity requirements (6, 7). As a result, there is an urgent requirement for a risk stratification model that is both more robust and more customized. This model should be able to assist doctors in distinguishing stroke from other causes of vertigo in a timely manner.

The most recent advancements in predictive modeling, which include methods for machine learning, have shown that they have the potential to support improvements in risk prediction across a range of clinical domains. This is something that has been proved by recent innovations in predictive modeling. To be more specific, multivariable models that take into account a wide variety of demographic, clinical, and diagnostic factors have the potential to produce more accurate predictions than traditional scoring systems. A bigger number of variables are incorporated into these models, which is the reason for this result twelve (8–10). On the other hand, the adoption of such models in clinical practice requires extensive internal validation in order to guarantee their dependability and generalizability. This is necessary in order to ensure that the models become reliable (11–13).

The aim of this study is to develop and internally validate a multivariable prediction model for stroke risk stratification in patients with acute persistent vertigo. We also compare the performance of our model with existing clinical scores to determine its potential advantage in clinical decision-making.

## Methods

### Study design and setting

This retrospective, single-center observational study was conducted at a tertiary medical center with specialized departments, including neurology and psychiatry, enrolling patients with acute persistent vertigo who underwent neurological evaluation and management. The study aimed to develop and internally validate a multivariable prediction model for stroke risk in patients presenting with acute persistent vertigo. Data were retrospectively collected from patients who presented between January 1, 2016 and December 31, 2024. Ethical approval was obtained from the local institutional ethics committee (Approval No.2025-KY007-01). Given the retrospective design of the study, the requirement for written informed consent was waived.

### Sample size calculation

To ensure the statistical power of the study, the sample size was calculated using an effect size of 0.5, assuming an alpha level of 0.05 and a power of 0.80. Based on this, a total of 689 participants were needed. The study successfully met the required sample size.

### Participants

Eligible patients were adults with acute persistent vertigo who underwent neurological evaluation and management at the tertiary medical center. The inclusion criteria were: Because the data-collection period spanned 9 years, calendar year of presentation was recorded and considered in sensitivity analyses to evaluate potential temporal variation in vascular risk-factor profiles and diagnostic practice. The main associations were materially unchanged after calendar year was included as an additional covariate, suggesting that temporal variability did not materially affect the final model.

At least eighteen years of age.

- A vertigo that lasts for longer than twenty-four hours.
- Accomplish a comprehensive clinical evaluation, which may include neuroimaging using a CT or MRI to confirm the diagnosis of stroke.

The following were the criteria for exclusion:

- A history of ischemic stroke or transient ischemic attack (TIA) that is known to have occurred.

The causes of dizziness that are not related to the cardiovascular system include benign paroxysmal positional vertigo (BPPV), vestibular neuritis, and Menière’s illness.

The patients who were unable to undergo neuroimaging owing to claustrophobia, contrast allergies, or any other factor that prevented them from doing so.

- Severe comorbid conditions with a National Institute of Health-related Services Score (NIHSS) of five or higher, which may have an impact on the clinical interpretation of the data.

**Figure 1:**
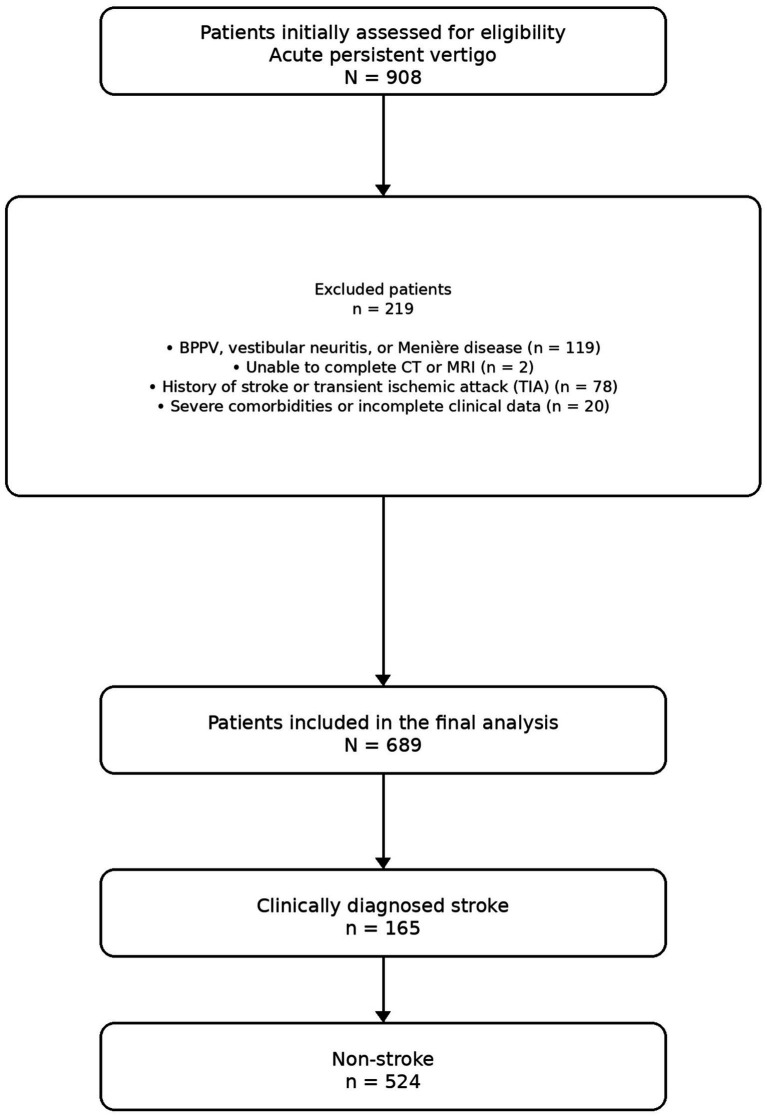
Flowchart of patient selection. Flowchart showing the selection of patients with acute persistent vertigo included in this study. Of 908 patients initially assessed for eligibility, 219 were excluded and 689 were included in the final analysis. Among the included patients, 165 were diagnosed with stroke and 524 were classified as non-stroke. BPPV, benign paroxysmal positional vertigo; CT, computed tomography; MRI, magnetic resonance imaging; TIA, transient ischemic attack.

The patient selection process is summarized in Figure 1.

### Data collection

Information was obtained from the electronic medical records of patients, including the following:

Demographic data included age, sex, body mass index (BMI), smoking status, and alcohol consumption.
A history of hypertension, diabetes, hyperlipidemia, coronary artery disease, atrial fibrillation, and strokes that have occurred in the past is included in the clinical information obtained from the patient.
Tinnitus, nausea, and vomiting are some of the symptoms that are present. Additionally, neurological findings that are associated with the illness, such as deficits in motor or sensory function, are often present.
Clinical evaluation scores can be broken down into three categories: the CNS score, the ABCD2 score, and the Triage-Plus score.

The findings of the neuroimaging examination, which include the site of the stroke and the kind of stroke (ischemic versus hemorrhagic), confirm the diagnosis of a stroke.

### Outcome measures

It was determined that the primary endpoint of the trial was the diagnosis of acute ischemic stroke, which was confirmed by CT or MRI within 14 days after the onset of symptoms. During the 30-day follow-up period, secondary outcomes which included neurological worsening and stroke recurrence were seen.

For clarity and reproducibility, the variables actually collected and analyzed were age, sex, body mass index, smoking status, alcohol consumption, hypertension, diabetes mellitus, hyperlipidemia, coronary heart disease, history of atrial fibrillation, previous stroke or transient ischemic attack, vertigo duration, nausea/vomiting, tinnitus, motor or sensory neurological deficits, ABCD2 score, CNS score, Triage-Plus score, and CT/MRI findings including stroke location and stroke type.

Age was analyzed as a continuous variable in the prediction model; therefore, the phrase “older age” refers to increasing age per year rather than to a prespecified age threshold. For descriptive reporting, age was summarized as mean ± standard deviation, and the overall cohort included 689 patients with a mean age of 60.3 ± 12.9 years, including 376 males (54.6%) and 313 females (45.4%).

Smoking was defined as current or former tobacco smoking documented in the electronic medical record and was coded as a binary variable. Pack-year information was reviewed when available, but it was not sufficiently complete for inclusion as a quantitative predictor.

Hyperlipidemia was defined as a documented clinical diagnosis of dyslipidemia, current lipid-lowering therapy, or an abnormal lipid profile recorded during the index encounter, including elevated total cholesterol, triglycerides, or low-density lipoprotein cholesterol, or reduced high-density lipoprotein cholesterol.

History of atrial fibrillation was defined as physician-diagnosed atrial fibrillation documented before or during the index evaluation, including paroxysmal, persistent, or permanent atrial fibrillation. A single unverified symptom description without electrocardiographic or medical-record confirmation was not sufficient for classification.

Nausea/vomiting was recorded as a binary symptom present at the initial presentation. Because nausea and vomiting are common in acute vertigo, they were not interpreted as specific markers of stroke in isolation; rather, they were evaluated only as part of the multivariable clinical context.

Tinnitus was recorded as present or absent at presentation. When available, laterality, pulsatile or non-pulsatile quality, and associated hearing loss were reviewed; however, these features were not consistently documented across the retrospective cohort and therefore were not entered as separate model predictors.

The CNS score was calculated according to the previously published clinical score originally developed for patients presenting with vertigo or dizziness, as described by Menekşe et al. (1)

## Statistical analysis

### Variable selection and model development

This study constructed a multivariable logistic regression model with the purpose of predicting the likelihood of stroke in patients who were experiencing acute persistent vertigo. For the purpose of feature selection, LASSO regression, which stands for least absolute shrinkage and selection operator, was utilized. Because it helps reduce the likelihood of overfitting and choose factors that are most strongly connected with the result, this strategy was selected. The variables that were incorporated into the final model were chosen through consideration of their clinical value and statistical significance. If the *p*-value was less than 0.05, then the inclusion of the predictor was regarded to be statistically significant.

### Hyperparameter tuning

For the purpose of determining the ideal regularization parameter (*λ*) for the LASSO regression model, hyperparameter tuning was carried out by means of 10-fold cross-validation. It was done in order to improve the performance of the model. With the use of the cross-validated area under the curve (AUC), a grid search was conducted across a range of *λ* values. The performance of the model was evaluated at each step by utilizing the AUC. The ideal value of λ was determined by calculating the value that minimized the error generated by cross-validation and maximized the performance of the model. This allowed us to pick the ideal value of *λ*. By using this approach, not only is it ensured that the model does not overfit or underfit the data, but it also chooses the regularization strength that is the most effective and efficient.

### Internal validation

The process of carrying out the internal validation was accomplished by the utilization of a 5-fold cross-validation. The decision was made to divide the dataset into five subsets, with four of those subsets being utilized for training purposes and the fifth subset being utilized for testing purposes. This technique was carried out five times in order to ensure that it is reliable and accurate. For the aim of determining the confidence intervals for model parameters with a 95% level of certainty, bootstrap resampling with a sample size of 1,200 was applied. For the purpose of ensuring that the model was stable and could be generalized, this was done.

In addition, we conducted sensitivity tests in order to evaluate the efficacy of the model taking into account a number of different assumptions (for example, reducing the threshold for stroke diagnosis).

### Model performance and evaluation

The effectiveness of the model was evaluated by employing a variety of significant metrics, which included the following:

An evaluation of the model’s ability to differentiate between patients who had suffered a stroke and those who had not suffered a stroke was carried out by calculating the area under the receiver operating characteristic curve (AUC). This was done in order to determine the level of discrimination that the model possessed.
The calibration was examined by using a calibration plot, which provided a comparison between the anticipated probability and the observed stroke outcomes. This was done in order to evaluate the calibration. The accuracy of the model was evaluated by computing both the intercept and the slope. Both of these quantities were computed.

*Score of Brier*: The Brier score is a statistic that is used to quantify the degree to which probabilistic forecasts are accurate. It is more likely that the performance is higher when the numbers are lower.

*Stratification of Risk*: Patients were divided into low, moderate, and high-risk groups according to the tertiles of their projected stroke probabilities that were used for stratification. We examined the rates of stroke events that occurred in each of the groups in order to evaluate the clinical utility of the model.

### Comparison with existing scoring systems

In order to determine the extent of our model’s contribution, we compared its performance to that of three well-established models for predicting the risk of stroke:

The score for ABCD2; The CNS score; The score on the Triage-Plus. Using the area under the curve (AUC), sensitivity, specificity, and Youden’s index, we compared the models. These measures were selected because of their extensive application in clinical practice for the purpose of assessing the risk of stroke in patients who are experiencing acute vertigo.

### Statistical software

Programming language Python, version 3.11.1, was utilized for each and every statistical analysis. In order to perform LASSO regression, the “sklearn” library was utilized. On the other hand, the “pandas,” “numpy,” and “matplotlib”libraries were utilized for data manipulation, statistical analysis, and visualization, respectively. Internal validation and hyperparameter tweaking were accomplished through the utilization of the “scikit-learn” package, wherein we employed 5-fold cross-validation and grid search techniques. Statistical significance was established at a level of *p* < 0.05. The handling of missing data was accomplished through the use of multiple imputation approaches in order to prevent bias in parameter estimates.

### Clinical application of the model

This prediction model, after it has been built and validated, may be utilized in clinical practice to assist physicians in risk stratification of patients who are experiencing acute persistent vertigo. Individualized stroke risk stratification is provided by the model, which classifies individuals into low, middle, and high-risk groups with similar characteristics. It is possible for this to serve as a decision-making guide in the therapeutic setting by identifying individuals who are at a high risk and could potentially benefit from rapid neuroimaging or prompt intervention. For instance, patients who are classified as high-risk can be given priority for stroke evaluation, but patients who are considered to be low-risk might not require immediate imaging. This may allow more efficient allocation of neuroimaging and specialist neurological resources within tertiary medical centers. In addition, the model can be incorporated into electronic health record systems to provide real-time assessment of stroke risk during clinical evaluation and management.

## Results

### Study population

A total of 689 consecutive patients with acute persistent vertigo were included in the study, of whom 165 (23.9%) were diagnosed with clinically confirmed stroke and 524 (76.1%) were classified as non-stroke. The mean age of the participants was 60.3 ± 12.9 years. The cohort included 376 males (54.6%) and 313 females (45.4%). The median duration of vertigo at presentation was 36 h (IQR 24–48 h). The patient selection process is shown in Figure 1, and the demographic and clinical characteristics of the study population, including the distribution of vascular risk factors and clinical symptoms, are summarized in Table 1.

**Table 1:** Baseline characteristics by clinical stroke diagnosis.

Variable	Stroke (*n* = 165)	Non-stroke (*n* = 524)	*p* value
Age, years	64.97 ± 14.57	58.82 ± 12.01	1.59e−06
Body mass index, kg/m^2^	25.29 ± 3.20	25.42 ± 3.10	0.662
CNS score	24.57 ± 3.63	19.45 ± 3.55	1.12e−40
ABCD2 score	7.30 ± 2.20	6.27 ± 1.86	1.33e−07
Triage-Plus score	3.35 ± 1.03	3.48 ± 1.05	0.187
Male sex	89 (53.9%)	287 (54.8%)	0.922
Alcohol drinking	55 (33.3%)	180 (34.4%)	0.884
Smoking	111 (67.3%)	316 (60.3%)	0.13
Hypertension	78 (47.3%)	102 (19.5%)	2.77e-12
Hyperlipidemia	56 (33.9%)	99 (18.9%)	8.51e−05
Diabetes mellitus	29 (17.6%)	36 (6.9%)	7.81e−05
Coronary heart disease	30 (18.2%)	28 (5.3%)	5.2e−07
History of atrial fibrillation	18 (10.9%)	16 (3.1%)	0.000115
Family history of stroke	15 (9.1%)	67 (12.8%)	0.254
History of central vertigo	61 (37.0%)	206 (39.3%)	0.655
Rotatory/visual symptoms	105 (63.6%)	370 (70.6%)	0.111
Nausea or vomiting	60 (36.4%)	167 (31.9%)	0.329
Tinnitus	41 (24.8%)	188 (35.9%)	0.0115
Posture-related symptoms	92 (55.8%)	289 (55.2%)	0.963

Continuous variables are presented as mean ± SD; categorical variables as *n* (%). p values: Welch’s t-test for continuous variables and chi-square test for categorical variables.

### Predictor selection

**Figure 2:**
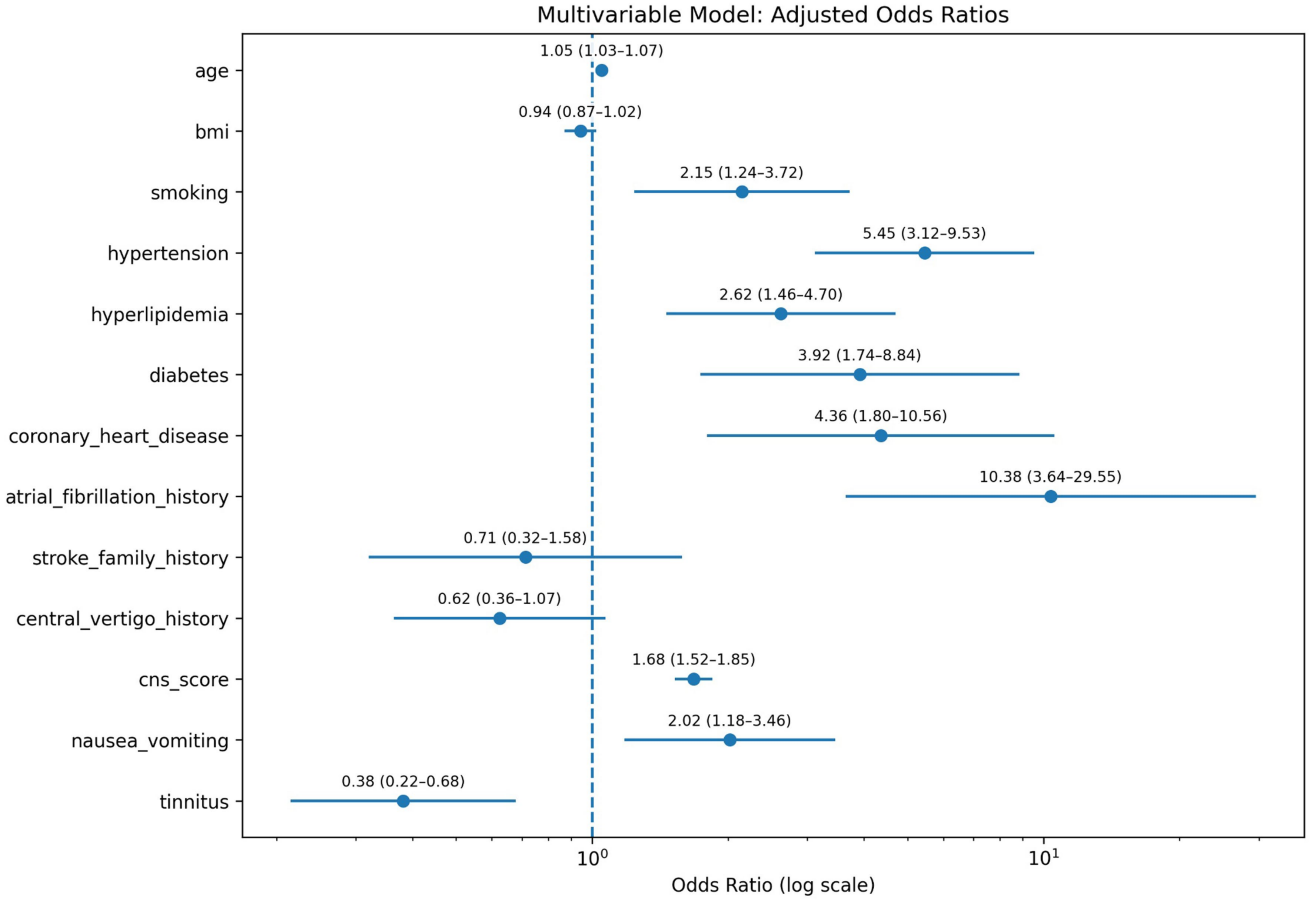
Forest plot of adjusted odds ratios in the multivariable logistic regression model. Forest plot showing adjusted odds ratios and 95% confidence intervals for predictors included in the final multivariable logistic regression model. The vertical dashed line indicates an odds ratio of 1.0.

The LASSO-regularized logistic regression identified significant predictors of stroke risk in acute persistent vertigo (Table 2). These included older age analyzed as a continuous variable (OR 1.050 per 1-year increase), smoking (OR 2.147), hypertension (OR 5.452), hyperlipidemia (OR 2.621), diabetes mellitus (OR 3.918), coronary heart disease (OR 4.361), history of atrial fibrillation (OR 10.376), higher CNS score (OR 1.679 per point), and nausea/vomiting (OR 2.020). Tinnitus was inversely associated with the probability of stroke (OR 0.382). The adjusted odds ratios and 95% confidence intervals of the final multivariable model are presented in Figure 2, and the detailed regression results are provided in Supplementary Table S2.

**Table 2:** Univariable and multivariable logistic regression for clinical stroke diagnosis.

Predictor	Univariable OR (95% CI)	*P* value	Multivariable OR (95% CI)	*P* value
Age, years	1.039 (1.024–1.055)	1.73e-07	1.050 (1.029–1.072)	2.51e−06
Body mass index, kg/m^2^	0.987 (0.934–1.044)	0.656	0.943 (0.870–1.022)	0.154
Smoking	1.353 (0.935–1.957)	0.109	2.147 (1.240–3.718)	0.00639
Hypertension	3.709 (2.551–5.394)	6.78e-12	5.452 (3.118–9.534)	2.71e−09
Hyperlipidemia	2.206 (1.494–3.256)	6.88e-05	2.621 (1.461–4.705)	0.00124
Diabetes mellitus	2.891 (1.710–4.885)	7.34e-05	3.918 (1.737–8.840)	0.001
Coronary heart disease	3.937 (2.273–6.816)	1e-06	4.361 (1.800–10.563)	0.0011
History of atrial fibrillation	3.888 (1.934–7.813)	0.000137	10.376 (3.643–29.554)	1.18e−05
Family history of stroke	0.682 (0.378–1.230)	0.203	0.712 (0.320–1.582)	0.404
History of central vertigo	0.905 (0.631–1.300)	0.59	0.624 (0.364–1.071)	0.087
CNS score	1.522 (1.416–1.636)	5.77e-30	1.679 (1.525–1.849)	6.45e−26
Nausea or vomiting	1.222 (0.847–1.762)	0.285	2.020 (1.180–3.458)	0.0104
Tinnitus	0.591 (0.398–0.878)	0.00916	0.382 (0.215–0.678)	0.00101

Predictors in the multivariable model were selected by LASSO-regularized logistic regression. OR, odds ratio; CI, confidence interval.

### Model performance

**Figure 3:**
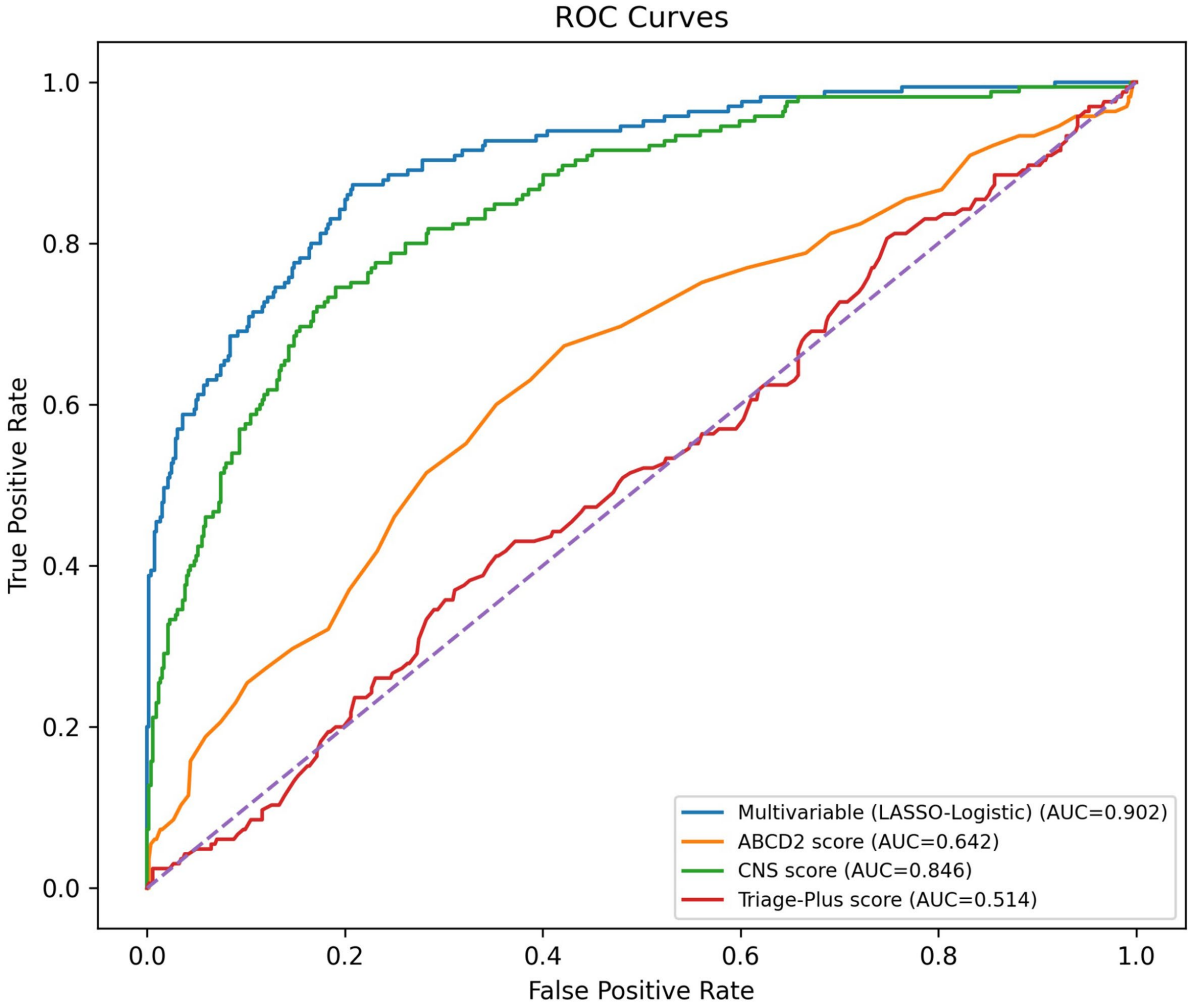
Receiver operating characteristic curves of the multivariable model and comparator scores. Receiver operating characteristic (ROC) curves comparing the discrimination performance of the multivariable model with that of the ABCD2 score, CNS score, and Triage-Plus score for identifying stroke in patients with acute persistent vertigo. AUC, area under the curve.

**Figure 4:**
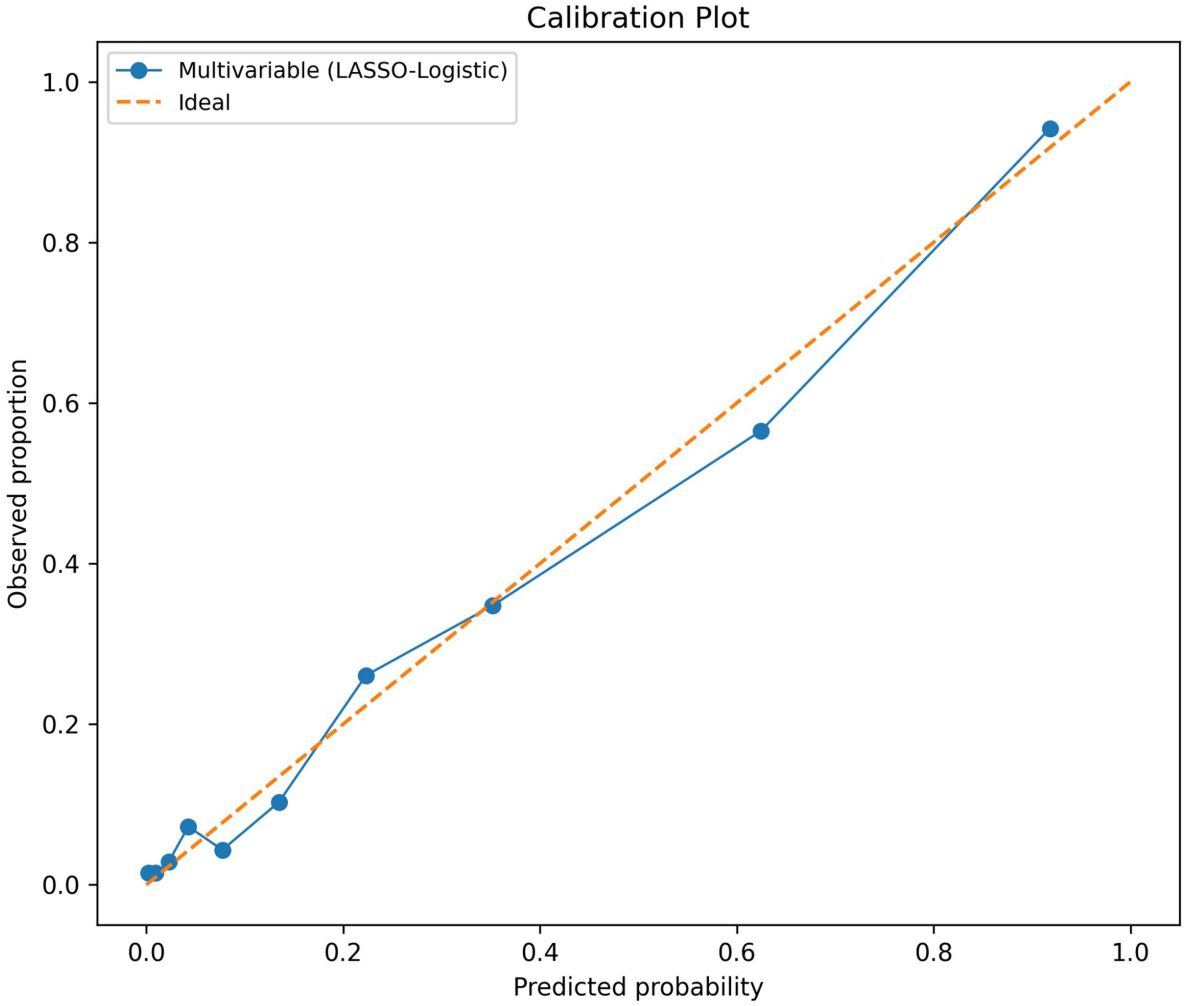
Calibration plot of the multivariable model. Calibration plot showing the agreement between predicted probabilities and observed stroke proportions for the multivariable model. The dashed line represents ideal calibration.

**Figure 5:**
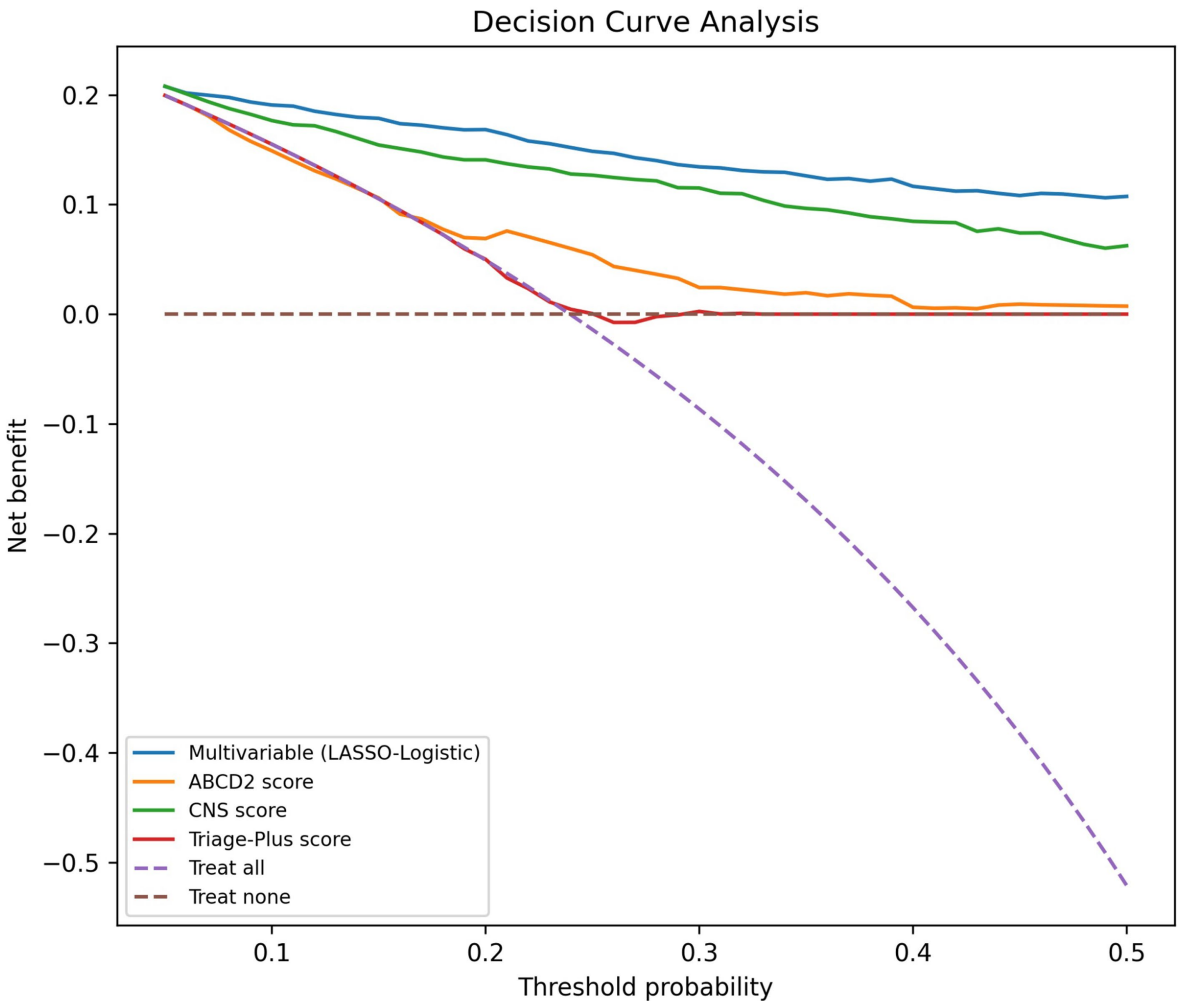
Decision curve analysis of the multivariable model and comparator scores. Decision curve analysis showing the net benefit of the multivariable model, ABCD2 score, CNS score, and Triage-Plus score across a range of threshold probabilities. The “treat all” and “treat none” strategies are shown as reference lines.

The multivariable logistic regression model showed excellent discrimination, with an area under the curve (AUC) of 0.902 (95% CI 0.874–0.927). At the optimal Youden threshold, the model achieved a sensitivity of 0.873 and a specificity of 0.792. The receiver operating characteristic curves are shown in Figure 3. Calibration analysis demonstrated good agreement between predicted and observed probabilities, with an intercept of −0.036 and a slope of 0.973, as illustrated in Figure 4. The model also showed favorable clinical utility on decision curve analysis (Figure 5). In addition, the Brier score was 0.096, indicating good overall predictive accuracy. A summary of model performance is presented in Table 3. As a supplementary evaluation of model performance, the precision-recall curve showed an average precision of 0.805, as shown in Supplementary Figure S1.

**Table 3:** Discrimination, calibration, and overall performance (internal validation via 5-fold CV with bootstrap CIs).

Model	AUC	AUC (95% CI)	Sensitivity (Youden)	Specificity (Youden)	Brier score	Calibration intercept	Calibration slope
Multivariable (LASSO-Logistic)	0.902	0.902 (0.874–0.927)	0.873	0.792	0.096	−0.036	0.973
ABCD2 score	0.642	0.642 (0.591–0.691)	0.673	0.578	0.172	−0.045	0.959
CNS score	0.846	0.846 (0.812–0.879)	0.745	0.809	0.123	−0.017	0.980
Triage-Plus score	0.514	0.514 (0.465–0.563)	0.412	0.647	0.182	−0.763	0.339

AUCs are computed from out-of-fold predicted probabilities; 95% CIs from bootstrap resampling (*n* = 1,200). Calibration intercept and slope were estimated by regressing the outcome on the logit of predicted probabilities.

### Comparison with existing scoring systems

The performance of the newly developed model was compared with three existing stroke risk assessment tools: the ABCD2 score, CNS score, and Triage-Plus score. The AUC was 0.642 for the ABCD2 score, 0.846 for the CNS score, and 0.514 for the Triage-Plus score. In contrast, the developed multivariable model achieved the highest AUC of 0.902, as shown in Figure 3. Detailed comparisons of discrimination and calibration performance are provided in Table 3.

### Risk stratification and validation

**Figure 6:**
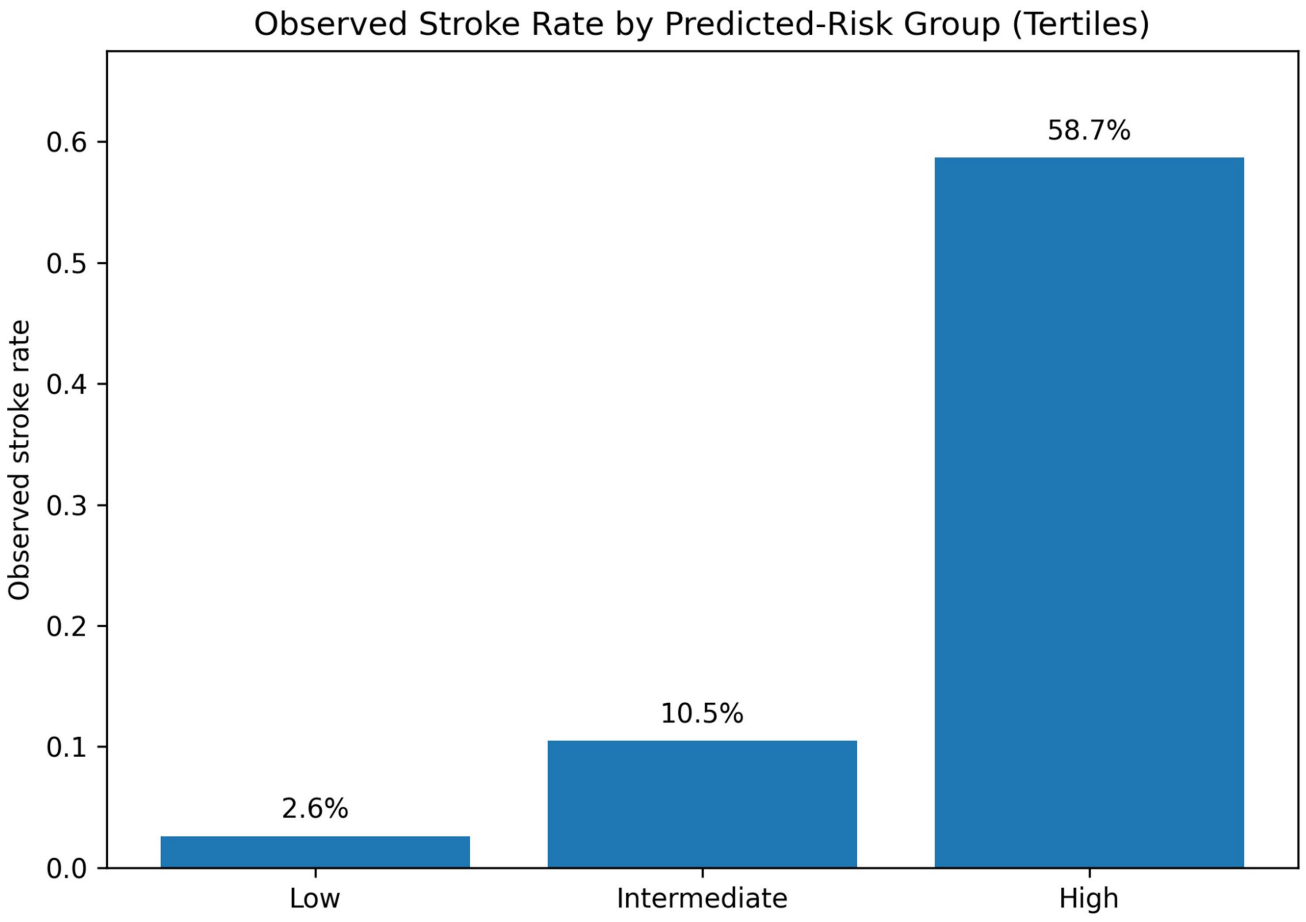
Observed stroke rates across predicted-risk groups. Bar chart showing observed stroke rates in the low-, intermediate-, and high-risk groups defined according to tertiles of predicted probabilities from the multivariable model.

Risk stratification was performed by dividing patients into three groups according to tertiles of predicted stroke probabilities. The tertile cut-off points for risk stratification were 0.039 and 0.242 (Table 4). The observed stroke event rates were 2.6, 10.5, and 58.7% in the low-, intermediate-, and high-risk groups, respectively (Table 4). The observed stroke rates across these predicted-risk groups are further illustrated in Figure 6, demonstrating the model’s ability to discriminate clinically meaningful risk categories.

**Table 4:** Risk stratification based on predicted probabilities from the multivariable model (tertiles).

Risk group (tertiles)	N	Stroke events	Event rate	Median predicted risk	P25–P75 predicted risk
Low	230	6	2.6%	0.012	0.004–0.023
Intermediate	229	24	10.5%	0.101	0.062–0.156
High	230	135	58.7%	0.560	0.344–0.851

Risk groups were defined by tertiles of predicted probability from the multivariable model (cut-points: 0.039 and 0.242).

### Clinical application of the model

In clinical practice, the stroke risk stratification model may help guide neurological risk assessment and diagnostic decision-making. Patients in the high-risk category, with a predicted probability greater than 0.242, may be prioritized for urgent neuroimaging or intervention, whereas patients in the low-risk group, with a predicted probability lower than 0.039, may not require immediate imaging. This model could also be integrated into electronic health record systems for real-time stroke risk assessment and more efficient resource allocation. These application scenarios remain conceptual and require prospective impact evaluation before routine implementation.

## Discussion

### Model performance and strengths

We created and internally validated a multivariable prediction model for the purpose of stratifying stroke risk in patients who presented with acute persistent vertigo. This study was conducted to investigate the topic. With an area under the curve (AUC) of 0.902 (95% confidence interval [CI]: 0.874–0.927), our model displayed outstanding performance, demonstrating excellent discriminating between stroke patients and those who did not have a stroke. The model contained clinical characteristics that are commonly available in clinical practice, such as age, smoking status, hypertension, hyperlipidemia, and atrial fibrillation. These variables were added into the model (14, 15). These findings suggest that our model may be useful in acute clinical settings where timely neurological evaluation and diagnostic decision-making are required (16).

The ABCD2 score, the CNS score, and the Triage-Plus score are all examples of stroke prediction techniques that are currently available; nonetheless, our model regularly outperformed these other tools. Despite the fact that our model produced a significantly stronger discriminatory ability, the ABCD2 score in our sample showed an area under the curve (AUC) of only 0.642. This suggests that our approach has significant clinical potential, particularly for patients who present with acute vertigo, which is a condition for which standard scoring systems have limits (1, 17).

The fact that our model has been calibrated correctly, as shown by the calibration plot (Figure 4), indicates that the stroke probabilities that were predicted are in good agreement with the events that really actually occurred. Further evidence that the model has a high level of prediction accuracy is provided by the Brier score of 0.096. According to these findings, our model has the potential to be utilized in the process of guiding clinical decision-making and improving patient outcomes in patients who are experiencing acute vertigo and are at a high risk for stroke (16, 18, 19).

### Clinical utility and application

A notable advantage of our stroke prediction model is that it has the potential to be utilized in a wide variety of clinical contexts. This is one of the most significant advantages of our model. Our model’s capacity to assist clinicians in identifying high-risk patients who require rapid imaging or intervention is especially helpful in acute neurological settings at tertiary medical centers, where timely diagnostic decision-making is important (20). It is possible, for instance, that patients who are classified as high-risk by the model (with a predicted chance that is greater than 0.242) would be given priority for rapid imaging, whereas patients who are classified as low-risk (with a predicted probability that is less than 0.039) might not require immediate examination.

The utilization of resources can be optimized and the stress on healthcare systems can be alleviated by the utilization of this stratification strategy, which can reduce the amount of unneeded imaging in patients who are at low risk (21). The incorporation of the model into electronic health record (EHR) systems would allow for automated real-time stroke risk assessment during clinical evaluation and management. In addition, the supplementary precision-recall curve provides further information on model performance in identifying stroke cases, with an average precision of 0.805 (Supplementary Figure S1).

Furthermore, in comparison to conventional scoring methods, our model provides unique risk assessments that are based on the clinical profile of each individual patient. This makes our model a more individualized approach. The precision and timeliness of clinical choices might be considerably improved as a result of this, which would ultimately lead to improvements in patient care and outcomes.

### Limitations and potential biases

Even if our model has a promising performance, there are a few constraints that need to be taken into consideration. In the first place, because this was a retrospective observational study, the findings can be affected by selection bias. This is especially true when considering the characteristics of patient recruitment and data collecting. The utilization of electronic health records may result in the introduction of problems such as missing data, which, despite being handled by multiple imputation, may still have an effect on the generalizability of the model (22).

In addition, our research was conducted at a single center with a particular patient population, which may not be representative of all individuals who suffer from acute persistent vertigo across a variety of clinical settings. In order to verify the model’s generalizability and usefulness across a variety of healthcare settings, it is necessary to conduct an external validation of the model using bigger cohorts that are comprised of multiple centers.

Another disadvantage is that imaging biomarkers and other modern diagnostic methods were not included in the construction of the model. Even though clinical variables had a significant predictive value, the incorporation of advanced imaging techniques, such as magnetic resonance imaging (MRI) diffusion-weighted imaging (DWI) or computed tomography (CT) perfusion imaging, could improve the accuracy of the model in identifying early ischemic changes, which are frequently observed in stroke patients who are experiencing acute vertigo.

### Future directions and research implications

A number of potential future directions are made possible as a result of the findings of this study. The next step that would make sense would be to do prospective validation of the model by employing separate cohorts in order to evaluate how well it performs in real-world situations. In order to guarantee that the model functions well across a wide range of patient groups and healthcare settings, this validation method would be of great assistance. Furthermore, the utilization of datasets from other institutions for the purpose of external validation has the potential to offer insights on the resilience and adaptability of the methodology.

When conducting additional study, it is important to evaluate the possibility of including advanced imaging biomarkers into the model. It is possible that the accuracy of the model could be improved by combining clinical predictors with imaging results such as DWI or CT perfusion. This would be especially beneficial in diagnosing early stroke in patients who present with equivocal clinical presentations with symptoms such as vertigo. It is possible that in the future, researchers will investigate the possibility of including genetic markers or the findings of laboratory tests (for example, inflammatory biomarkers) into the prediction model in order to improve its accuracy.

The investigation of the model’s influence on clinical outcomes is yet another significant direction that future research should take. In order to get significant insights into the practical utility of this model in real-world clinical practice, studies that evaluate how the utilization of this model effects clinical decision-making, resource allocation, and patient outcomes would be beneficial. In addition, we predict that the model may develop over time through the application of machine learning techniques. This would allow for the incorporation of continual updates when new clinical data and improved biomarkers become available.

## Conclusion

In conclusion, we have constructed and internally validated a reliable multivariable prediction model for the purpose of stratifying patients with acute persistent vertigo who are at risk for having a stroke. The model displayed outstanding performance, which was superior to the clinical stroke prediction models that were already in existence. This methodology has the potential to assist clinicians in making early decisions that are based on evidence, hence improving outcomes for patients who are experiencing acute vertigo. It does this by giving physicians with tailored stroke risk stratification. To verify the therapeutic usefulness of this model and assess how well it can be incorporated into standard clinical practice, it is necessary to do additional research and conduct external validation investigations.

## Data Availability

The raw data supporting the conclusions of this article will be made available by the authors, without undue reservation.

## Supplementary material

SUPPLEMENTARY FIGURE S1 Precision-recall curve of the multivariable model. Precision-recall curve showing the diagnostic performance of the multivariable LASSO-logistic model for stroke prediction in patients with acute persistent vertigo. The average precision was 0.805.
