# Supplementary material for: Risk stratification for stroke in acute persistent vertigo: development and internal validation of a multivariable prediction model
Source: Front Neurol. 2026 Jun 24;17:1822762. doi: 10.3389/fneur.2026.1822762 (PMC13341476; doi:10.3389/fneur.2026.1822762)
Supplement: Supplementary file 1 [file Table_1.DOCX]

**Supplementary Table S1. Variable definitions and coding**

| **Variable** | **Type** | **Coding** |
| --- | --- | --- |
| Age, years | Continuous | Original scale |
| Male sex | Binary | 0 = No, 1 = Yes |
| Body mass index, kg/m² | Continuous | Original scale |
| Alcohol drinking | Binary | 0 = No, 1 = Yes |
| Smoking | Binary | 0 = No, 1 = Yes |
| Hypertension | Binary | 0 = No, 1 = Yes |
| Hyperlipidemia | Binary | 0 = No, 1 = Yes |
| Diabetes mellitus | Binary | 0 = No, 1 = Yes |
| Coronary heart disease | Binary | 0 = No, 1 = Yes |
| History of atrial fibrillation | Binary | 0 = No, 1 = Yes |
| Family history of stroke | Binary | 0 = No, 1 = Yes |
| History of central vertigo | Binary | 0 = No, 1 = Yes |
| CNS score | Continuous | Original scale |
| ABCD2 score | Continuous | Original scale |
| Triage-Plus score | Continuous | Original scale |
| Rotatory/visual symptoms | Binary | 0 = No, 1 = Yes |
| Nausea or vomiting | Binary | 0 = No, 1 = Yes |
| Tinnitus | Binary | 0 = No, 1 = Yes |
| Posture-related symptoms | Binary | 0 = No, 1 = Yes |

Binary variables are coded as 0/1. Scores are used on their original scales.
