# Supplementary material for: Risk stratification for stroke in acute persistent vertigo: development and internal validation of a multivariable prediction model
Source: Front Neurol. 2026 Jun 24;17:1822762. doi: 10.3389/fneur.2026.1822762 (PMC13341476; doi:10.3389/fneur.2026.1822762)
Supplement: Supplementary file 2 [file Table_2.DOCX]

**Supplementary Table S2. Coefficients of the multivariable model (logistic regression)**

| **Predictor** | **β** | **SE** | **Adjusted OR (95% CI)** | **P value** |
| --- | --- | --- | --- | --- |
|  |  |  |  |  |
| Age, years | 0.0490 | 0.0104 | 1.050 (1.029–1.072) | 2.51e-06 |
| Body mass index, kg/m² | -0.0588 | 0.0412 | 0.943 (0.870–1.022) | 0.154 |
| Smoking | 0.7641 | 0.2802 | 2.147 (1.240–3.718) | 0.00639 |
| Hypertension | 1.6961 | 0.2851 | 5.452 (3.118–9.534) | 2.71e-09 |
| Hyperlipidemia | 0.9637 | 0.2984 | 2.621 (1.461–4.705) | 0.00124 |
| Diabetes mellitus | 1.3656 | 0.4151 | 3.918 (1.737–8.840) | 0.001 |
| Coronary heart disease | 1.4727 | 0.4514 | 4.361 (1.800–10.563) | 0.0011 |
| History of atrial fibrillation | 2.3395 | 0.5340 | 10.376 (3.643–29.554) | 1.18e-05 |
| Family history of stroke | -0.3396 | 0.4073 | 0.712 (0.320–1.582) | 0.404 |
| History of central vertigo | -0.4714 | 0.2755 | 0.624 (0.364–1.071) | 0.087 |
| CNS score | 0.5184 | 0.0492 | 1.679 (1.525–1.849) | 6.45e-26 |
| Nausea or vomiting | 0.7029 | 0.2743 | 2.020 (1.180–3.458) | 0.0104 |
| Tinnitus | -0.9631 | 0.2929 | 0.382 (0.215–0.678) | 0.00101 |

β coefficients are from the fitted multivariable logistic regression on the full dataset. Predictors were selected by LASSO.
