# Supplementary figures and images for: Risk stratification for stroke in acute persistent vertigo: development and internal validation of a multivariable prediction model
Source: Front Neurol. 2026 Jun 24;17:1822762. doi: 10.3389/fneur.2026.1822762 (PMC13341476; doi:10.3389/fneur.2026.1822762)

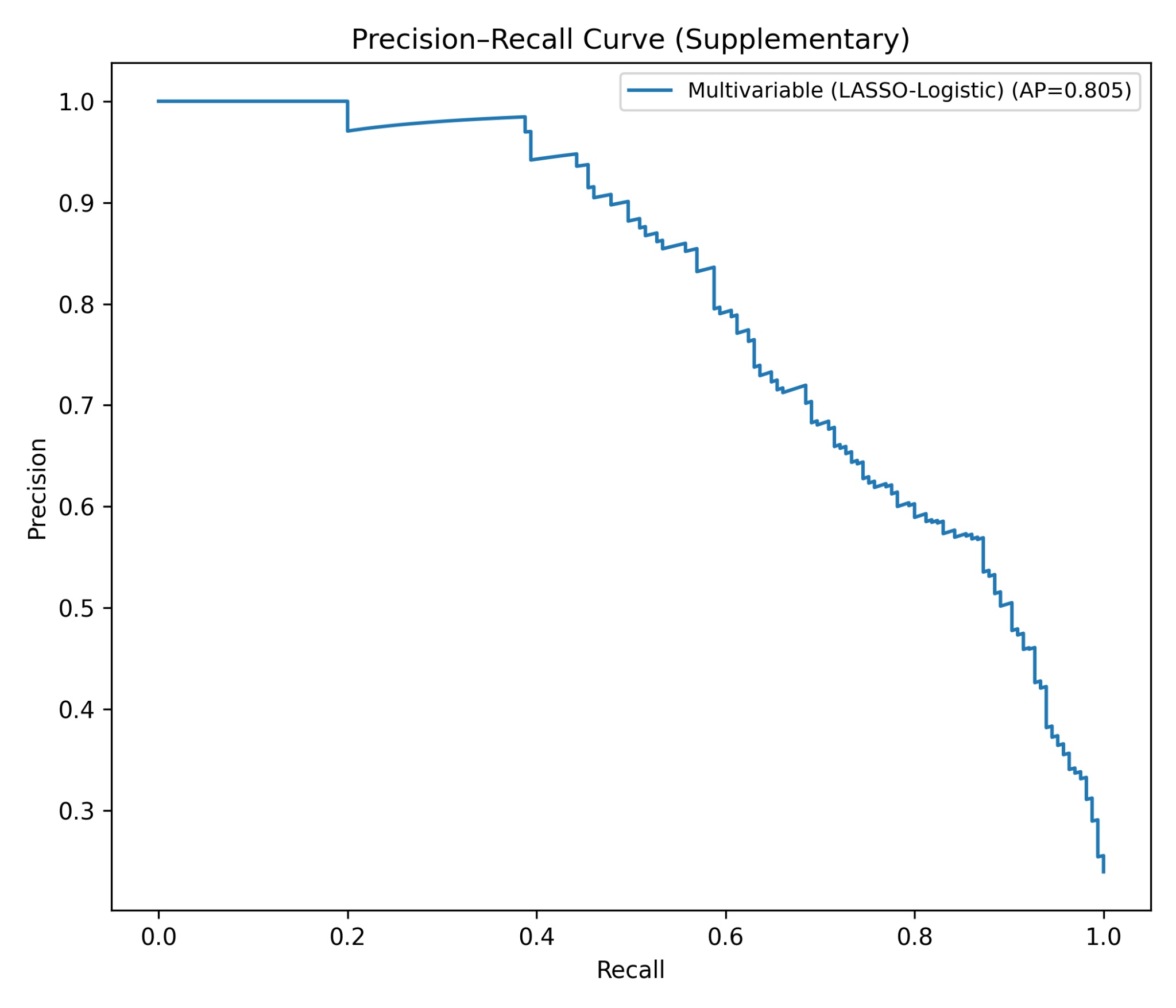

Supplement: SUPPLEMENTARY FIGURE S1 — Precision-recall curve of the multivariable model. Precision-recall curve showing the diagnostic performance of the multivariable LASSO-logistic model for stroke prediction in patients with acute persistent vertigo. The average precision was 0.805. [file Image_1.JPEG]
